# Loss of UHRF2 expression is associated with human neoplasia, promoter hypermethylation, decreased 5-hydroxymethylcytosine, and high proliferative activity

**DOI:** 10.18632/oncotarget.12583

**Published:** 2016-10-12

**Authors:** Huarui Lu, Sweta Bhoopatiraju, Hongbo Wang, Nolan P. Schmitz, Xiaohong Wang, Matthew J. Freeman, Colleen L. Forster, Michael R. Verneris, Michael A. Linden, Timothy C. Hallstrom

**Affiliations:** ^1^ Department of Pediatrics, University of Minnesota, Minneapolis, MN 55455, USA; ^2^ BioNet, Academic Health Center, University of Minnesota, Minneapolis, MN 55455, USA; ^3^ Department of Laboratory Medicine and Pathology, University of Minnesota, Minneapolis, MN 55455, USA

**Keywords:** ubiquitin-like with PHD and ring finger domains 2, UHRF2, 5-hydroxymethylcytosine, UHRF1, leukemia

## Abstract

Ubiquitin-like with PHD and ring finger domains 2 (UHRF2) binds to 5-hydroxymethylcytosine (5hmC), a DNA base involved in tissue development, but it is unknown how their distribution compares with each other in normal and malignant human tissues. We used IHC on human tumor specimens (160 from 19 tumor types) or normal tissue to determine the expression and distribution of UHRF2, Ki-67, and 5hmC. We also examined UHRF2 expression in cord blood progenitors and compared its expression to methylation status in 6 leukemia cell lines and 15 primary human leukemias. UHRF2 is highly expressed, paralleling that of 5hmC, in most non-neoplastic, differentiated tissue with low Ki-67 defined proliferative activity. UHRF2 is expressed in common lymphoid progenitors and mature lymphocytes but not common myeloid progenitors or monocytes. In contrast, UHRF2 immunostaining in human cancer tissues revealed widespread reduction or abnormal cytoplasmic localization which correlated with a higher Ki-67 and reduced 5hmC. UHRF2 expression is reduced in some leukemia cell lines, this correlates with promoter hypermethylation, and similar UHRF2 methylation profiles are seen in primary human leukemia samples. Thus, UHRF2 and 5hmC are widely present in differentiated human tissues, and UHRF2 protein is poorly expressed or mislocalized in diverse human cancers.

## INTRODUCTION

Epigenetic changes to DNA and histones control stem cell differentiation and the production of all types of the body's cells, and these processes often malfunction in human cancer. Methylation of cytosine (C) by DNA methyltransferase (DNMT) enzymes generates 5-methylcytosine (5mC) which is essential for transcriptional regulation and development. Cytosine methylation occurs most often at CpG dinucleotides, which form CpG islands (CGIs) when present together at very high density and which are often present in gene promoters. Methylation at CGIs is most often associated with gene silencing whereas demethylation of 5mC often leads to gene expression. 5mC marks can be converted to 5-hydroxymethylcytosine (5hmC) by the Ten-Eleven-translocation-2 (TET2) enzyme [1, 2]. This discovery has generated great interest because TET enzymes are frequently mutated in cancer, and the 5hmC mark is well documented as being lost in different cancer types relative to normal tissue [3, 4, 5, 6]. It is also becoming widely clear that 5hmC is involved in gene expression during tissue development [7, 8].

The UHRF2 protein was recently identified as having stronger binding affinity toward 5hmC than to C or 5mC, and independent biochemical analysis confirmed this finding [9, 10]. UHRF2 contains multiple domains, including a set- and ring- associated “SRA” domain that binds 5-hydroxymethylcytosine (5hmC), and a C-terminal Ring domain with E3 ubiquitin ligase activity [11–13]. By contrast, the SRA domain of the highly similar UHRF1 protein preferentially binds hemi-methylated DNA during replication and recruits DNMT1 to conserve methylation in the daughter strand [14–17]. UHRF2 and 5hmC were recently shown to co-localize at DNA by ChIP-seq analysis [18].

UHRF2 is a potential tumor suppressor gene located within the 9p24 chromosomal region which is among the most frequent sites of DNA copy number lost in human cancer [19, 20]. UHRF2 was one of 100 “candidate cancer (CAN) genes” mutated in breast or colorectal cancer at a higher frequency than background mutation frequency [21, 22]. UHRF2 has also been recovered by different research groups from diverse sleeping-beauty mutagenesis screens wherein its deletion promotes liver, colon, nervous system tumors and notably colorectal cancers with severe disease [23–25]. UHRF2 also controls gene expression relating to epithelial-mesenchymal transition and promotes cell invasion [26].

To learn more about the link between UHRF2 and 5hmC co-expression, we performed IHC using antibodies to compare their expression across a variety of normal and malignant human tissues. These analyses have generated several conclusions. First, UHRF2 and 5hmC are widely present in terminally differentiated cells. Second, UHRF2 protein levels are significantly reduced or mislocalized in a wide variety of human cancers compared to normal tissue. Finally, the UHRF2 promoter is heavily methylated in several leukemia cell lines and this correlates with reduced expression and similar methylation is also observed in human leukemia samples.

## RESULTS

We used immunohistochemistry (IHC) to examine the expression pattern of UHRF2 in a large number of different normal or malignant adult human tissues. We carefully validated the UHRF2 antibody for IHC using cell line FFPE blocks (Supplementary Figure S1) using cell lines that over- or under-express UHRF2. U2OS cells with wild-type UHRF2 stain strongly but the shUHRF2 cells do not. We also performed IHC on adjacently sectioned tissues with antibodies that recognize proliferating cells (Ki67) or the 5hmC base. This allowed us to determine if UHRF2 is expressed in differentiated and/or proliferating normal tissue and if it is expressed in or absent from Ki67 positive regions of tumor sections. The 5hmC antibody has been extensively validated for IHC use with formalin-fixed paraffin embedded (FFPE) tissues [3, 4, 27, 28]. Normal tissue was arranged into tissue microarrays (TMAs) so that staining with anti-UHRF2 antibodies was conducted simultaneously on normal, and tumor tissues. We first evaluated UHRF2 expression patterns in normal human tissues. Results shown are representative of at least three individual tissue stainings.

**Figure 1:**
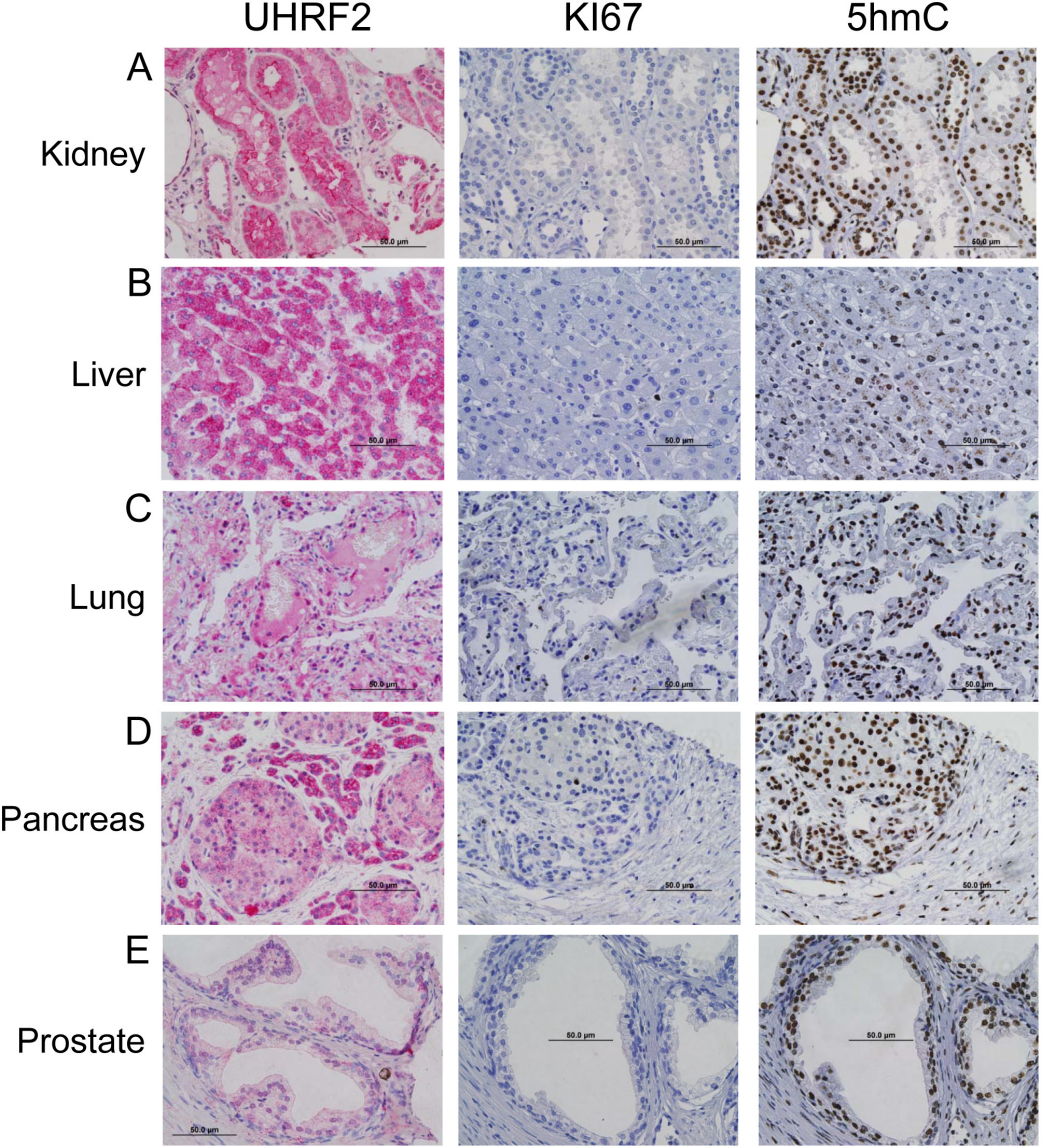
UHRF2 is expressed in differentiating cell populations IHC for UHRF2, Ki67, and 5hmC was performed on normal human tissue from tonsil, oral mucosa, jejunum, testis, and uterus.

We evaluated the UHRF2 staining pattern in normal, non-neoplastic adult differentiated tissue from kidney, liver, lung, pancreas, and prostate (Figure 1). Serially sectioned tissue was also processed for IHC using antibodies to detect Ki67 and 5hmC. The UHRF2 protein was detectable by IHC in each of these differentiated tissues, although staining is weaker in the lung and prostate than in kidney, liver, and pancreas. UHRF2 is generally present in both the nuclear and cytoplasmic compartments of these tissues. These tissues were all negative for Ki67 staining, suggesting a low proliferative activity. In contrast, they were each strongly positive for 5hmC staining. Staining patterns from many other adult differentiated tissues are listed in Table 1 and shown in Supplementary Figure S2A & S2B. An identical staining profile (UHRF2^+^, Ki67^−^, 5hmc^+^) was observed for numerous other adult differentiated non-neoplastic tissues, such as epididymis, fallopian tube, gallbladder, skeletal muscle, parathyroid, salivary gland, seminal vesicle, and thyroid. Thus, terminally differentiated cells in many organs, some of which have previously been demonstrated to be Ki67^−^/5hmC^+^, are generally all also positive for UHRF2.

**Table 1:** List of UHRF2 in different cell types of adult human tissue

Tissue	Figure	Cell Type
Adrenal Cortex	Supplementary Figure S2A	Fasiculata
Appendix	Supplementary Figure S3A	Germinal center
Cervix (Uterine)	Supplementary Figure S3B	Squamous epithelial
Colon	Supplementary Figure S3C	Differentiating villi
Epididymis	Supplementary Figure S2A	Some Principal & Basal
Esophageal Mucosa	Supplementary Figure S3B	Squamous epithelial
Fallopian Tube	Supplementary Figure S2A	Ciliated epithelial
Gall Bladder	Supplementary Figure S2A	Columnar epithelial
Ilium	Supplementary Figure S3C	Differentiating villi
Jejunum	Figure 2	Differentiating villi
Kidney	Figure 1	Distal/prox convoluted tubules
Liver	Figure 1	Hepatocytes
Lung	Figure 1	Alveolar
Oral Mucosa	Figure 2	Squamous epithelial
Ovary	Supplementary Figure S2B	Stroma and follicle
Pancreas	Figure 1	Islets of Langerhans
Parathyroid	Supplementary Figure S2B	Chief
Placenta	Supplementary Figure S2B	Decidual, trophoblastic, Hofbauer
Prostate	Figure 1	Cuboidal epithelial
Rectum	Supplementary Figure S3C	Differentiating villi
Salivary Gland	Supplementary Figure S2B	Interlobular ductal cells
Seminal Vesicle	Supplementary Figure S2B	Columnar epithelial & Lamina propria
Skeletal Muscle	Supplementary Figure S2A	Myocytes
Skin	Supplementary Figure S3B	Squamous epithelial
Spleen	Supplementary Figure S3A	Germinal center
Stomach	Supplementary Figure S3C	Differentiating villi
Testis	Figure 2	Spermatogonia, Spermatocytes, Sertoli
Thyroid	Supplementary Figure S2B	Folicular & Cuboidal epithelial
Tonsil	Figure 2	Germinal center
Uterus - Endometrial	Figure 2	Glandular epithelial
Vagina	Supplementary Figure S3B	Squamous epithelial

All tissues examined by IHC for UHRF2, Ki67 and 5hmC are listed, and the UHRF2 positive cell types are listed.

**Figure 2:**
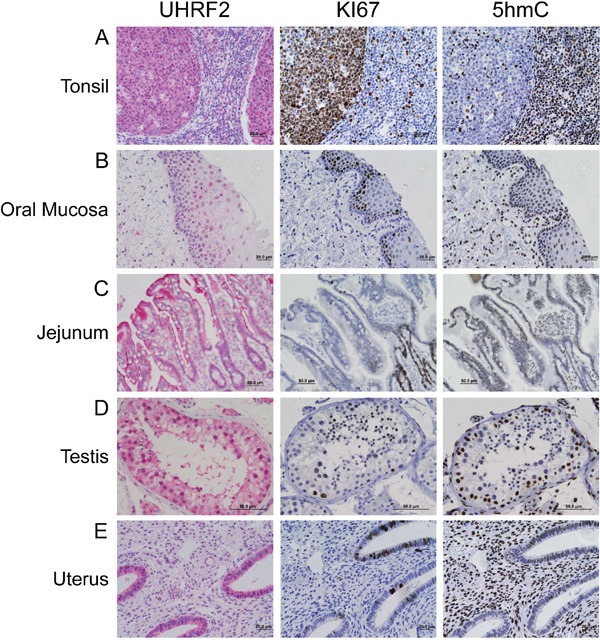
UHRF2 and 5hmC are hallmarks of terminally differentiated cells Adult human tissue from kidney, liver, lung, pancreas and prostate were analyzed by IHC for UHRF2, Ki67 and 5hmC levels.

Our staining of numerous different adult tissues also included several which contained Ki67^+^ progenitor cells in the process of differentiating. For example, UHRF2 strongly stained the germinal centers (GCs) of peripheral lymphoid organs including the tonsil (Figure 2), appendix and spleen (Supplementary Figure S3A). In contrast to adult differentiated tissues, which were all UHRF2^+^, Ki67^−^, 5hmC^+^, UHRF2 positive GCs were strongly Ki67 positive and 5hmC negative. The loss of 5hmC in GCs profile has been documented [29–31]. Intriguingly, the promoter region of the UHRF2 gene transitions from highly methylated in naïve B-lymphocytes to unmethylated in B-lymphocytes undergoing the GC reaction, consistent with the expression pattern we observe and suggestive of a potential role of UHRF2 in differentiating B-lymphocytes [32]. It also indicates that UHRF2 expression is not limited to terminally differentiated cells. We also observed UHRF2 expression in basal and suprabasal squamous epithelial cells of the oral mucosa (Figure 2), esophageal mucosa, skin, cervix and vagina (Table 1 and Supplementary Figure S3B). UHRF2 and 5hmC clearly stained Ki67 negative cells of the basal layer. Cells of the suprabasal layer were both UHRF2 positive and negative, and generally displayed strong nuclear localization of UHRF2 unlike the terminally differentiated cells which typically showed both nuclear and cytoplasmic localization. Most of these cells retained 5hmC in the suprabasal layer. The GI tract is a classical model of tissue differentiation where progenitor cells in the crypt produce differentiated cells of the villi. UHRF2 is present in both the proliferating and differentiated cells of the jejunum (Figure 2), colon, ileum, lower stomach, and rectum (Table 1 and Supplementary Figure S3C). In each of these tissue, Ki67 and 5hmC staining is mutually exclusive, with progenitor cells in the crypt (Ki67^+^/5hmC^−^) producing differentiated cells of the villi (Ki67^−^/5hmC^+^) [3, 31]. IHC staining of testis revealed spermatogonia with Ki67^−^ nuclei that stained positive for UHRF2 and 5hmC. Differentiating spermatids displayed evidence of loss of 5hmC staining, acquisition of Ki67^+^ staining and retention of nuclear UHRF2 staining (Figure 2D). UHRF2 also strongly stained endometrial glandular cells in the uterus, which contained Ki67 or 5hmC positive admixed cells (Figure 2E).

Having established the expression pattern of UHRF2 across numerous normal human tissues, we asked if and how the UHRF2 expression pattern is altered in human tumors originating from some of these tissues. In total, we examined 160 specimens derived from 19 different tumor types organized in tissue microarrays. This staining of tumor sample TMAs was conducted simultaneously with staining of normal tissue TMAs to reduce the likelihood of batch or slide variations in staining. This broad analysis revealed three main themes that describe the staining pattern of UHRF2 across a variety of human tumors. Some tumors retain similar expression and localization of UHRF2 compared to matching normal tissues. Second, UHRF2 appears abnormally localized to the cytoplasm but excluded from the nucleus in a subset of human tumors. Finally, UHRF2 frequently displays a significant reduction in expression in diverse tumor types. We show representative examples of each category from gastric, hepatocellular and testicular cancers.

**Figure 3:**
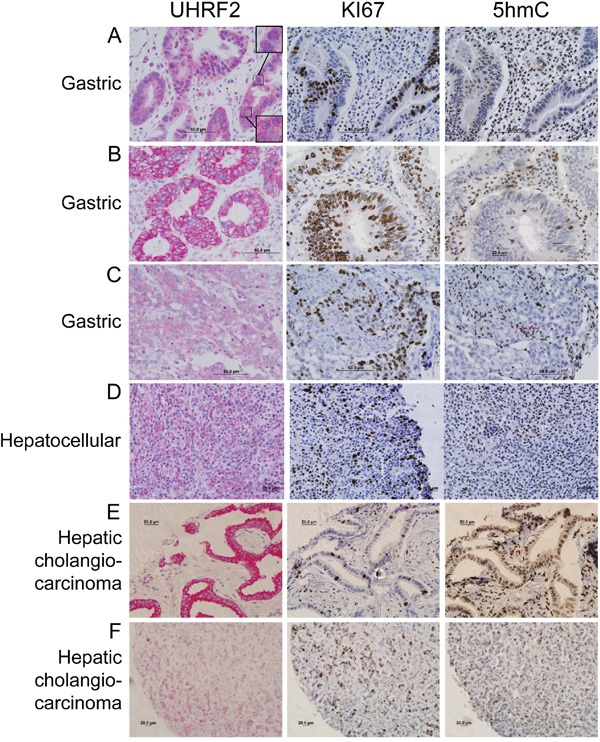
UHRF2 protein levels are mislocalized or reduced compared to normal tissue in gastric and hepatocellular human tumors Human cancer tissue was analyzed by IHC for UHRF2, Ki67, and 5hmC and displayed as representative micrographs.

UHRF2 expression was observed in the nucleus and cytoplasm of both proliferating and differentiating epithelial cells in the GI tract villi. Gastric tumors typically arise from these epithelial cells, and in some cases these tumors retain strong UHRF2 staining that is comparable to normal tissue (Figure 3A). We observed that gastric tumors also often display altered localization of UHRF2 predominantly in the cytoplasm but not the nucleus (Figure 3B). Similar staining was observed in some colorectal tumors in this study (Supplementary Figure S4A) and by Lu *et al*., who also reported cytoplasmic staining of UHRF2 in a large cohort of human colorectal tumors [33]. These gastric tumor cells were strongly Ki67 positive, and cells from the same region in serially sectioned tissue were notably lacking 5hmC staining. A final subset of gastric tumors displays a significant reduction in UHRF2 staining (Figure 3C). These tumors with reduced UHRF2 expression were highly Ki67^+^ and 5hmC^−^. We assessed UHRF2 staining across a sampling of hepatic cholangiocarcinomas, which arise from biliary ductular epithelial cells. Some of these tumors retained moderate levels of UHRF2 (Figure 3D). Another subset of these cancers showed a significant increase in cytoplasmic localization of UHRF2 (Figure 3E). A final fraction of cholangiocarcinomas showed sharply reduced levels of UHRF2 (Figure 3F). Testicular cancer is thought to arise from germ cells, and these cells displayed strong nuclear UHRF2 staining in normal tissue. We show an example of a highly proliferative, 5hmC^−^ testis cancer where UHRF2 levels are observed at moderate levels primarily in the nucleus (Supplementary Figure S4A). A second, unusual example, shows strong UHRF2 staining in a perinuclear pattern in cells that are generally Ki67^+^ and 5hmC^−^. Lastly, UHRF2 protein levels are also reduced in a highly Ki67+ testis cancer.

**Figure 4:**
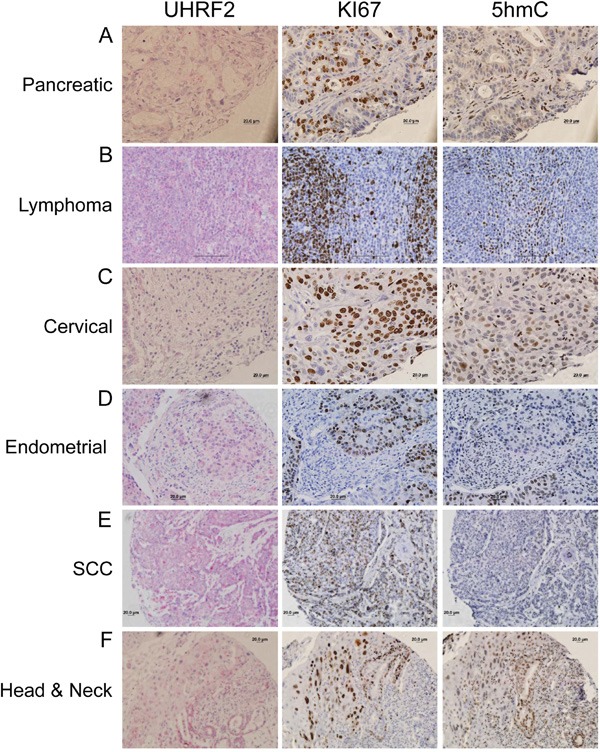
UHRF2 protein levels are reduced across numerous different human tumor types Human cancer tissue was analyzed by IHC for UHRF2, Ki67, and 5hmC and displayed as representative micrographs.

We sought to determine if loss of UHRF2 expression was a recurring theme in other human tumor types. We show examples of significant reduction in UHRF2 staining in pancreatic, lymphoma, cervical, endometrial, squamous cell carcinoma (SCC) and head & neck human tumors (Figure 4). UHRF2 staining was assessed in regions that were highly proliferative in serially sectioned tissue. Highly proliferative regions (Ki67^+^) almost always display reduced 5hmC levels by IHC staining, as seen previously in studies using human tumor samples. Occasional exceptions to this rule were noted. For example, staining of a testis cancer and a lung SCC (Supplementary Figure S4A) each contained >90% cells staining positive for Ki67 and 5hmC.

**Figure 5:**
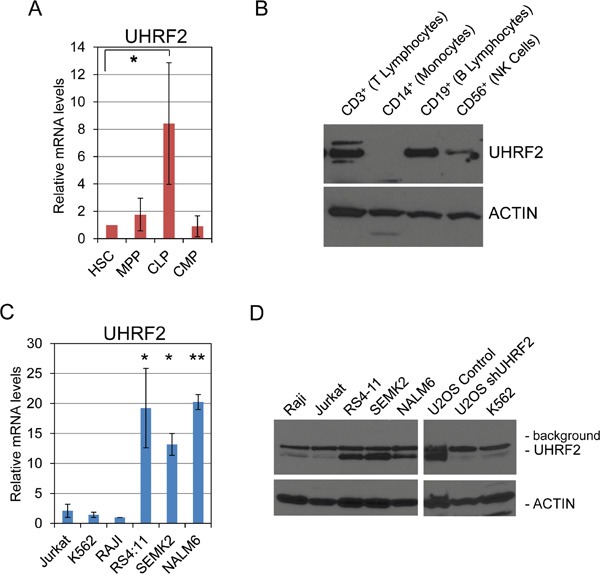
UHRF2 is expressed in human common lymphoid progenitors and mature B- and T- lymphocytes, and its mRNA and protein levels are substantially reduced in several human leukemia and lymphoma cells lines **A.** UHRF2 mRNA expression is increased in human common lymphoid progenitors (CLP) relative to hematopoietic stem cells (HSC), multipotent progenitors (MPP) and common myeloid progenitors (CMP). (n=3) **B.** UHRF2 protein is present in human B, T, and NK cells but missing from monocytes. **C.** UHRF2 mRNA expression is decreased in Jurkat (T-cell leukemia), K562 (lymphoblasts) and RAJI (Burkitt's lymphoma) but intact in the pro-B and pre-B cell lines RS4:11, SEMK2, and NALM6. **D.** UHRF2 protein is absent in Jurkat, K562, and RAJI cell lines but present in RS4:11, SEMK2, and NALM6. See band reduced from U2OS cells containing shUHRF2 vs control. *, p < 0.05; **, p < 0.01. Error bars represent standard deviation from the mean.

This analyses documented UHRF2 expression across a variety of normal and tumor solid tissues, but had not assessed the presence of UHRF2 in normal or malignant hematological cells, so we assessed relative levels of UHRF2 in hematopoietic stem cells, progenitors or differentiated cells. First, hematopoietic stem cells (HSCs), multipotent progenitors (MPPs), common lymphoid progenitors (CLPs), and common myeloid progenitors (CMPs) were separated by fluorescent activated cell sorting (FACS) from human umbilical cord blood. RNA was isolated and quantified, and qPCR was used to measure UHRF2 mRNA levels normalized to GAPDH (Figure 5). We found that UHRF2 levels were lowest in HSCs and remained comparably low in MPPs and CMPs. Surprisingly, UHRF2 mRNA was elevated around 8-fold in CLPs compared to HSCs. Next, mature lymphocyte populations were isolated from human blood and protein analyzed by immunoblotting. We observed comparable expression of UHRF2 in isolated B- and T-lymphocytes. UHRF2 levels are reduced but still present in CD56+ natural killer cells. We were unable to detect UHRF2 in CD14^+^ monocytes. Thus, UHRF2 appears to be predominantly expressed in mature cells of lymphoid origin and in CLPs, but not in the more undifferentiated HSCs or MPPs, or in cells of the myeloid lineage (CMPs, monocytes).

Our previous analysis of UHRF2 expression in normal vs. cancer tissue indicated that its levels are decreased in a wide variety of human tumor types. To determine if UHRF2 is also decreased in hematopoietic cancer cells, we analyzed UHRF2 mRNA and protein expression pattern across six different leukemia or lymphoma cell lines. mRNA was isolated from Jurkat (T-lymphoblastic leukemia), K562 (lymphoblasts), RAJI (Burkitt lymphoma), RS4:11 & SEMK2 (t(4;11)-positive precursor B-lymphoblastic leukemia), and NALM6 (B-lymphoblastic leukemia) cells and qPCR was utilized to compare UHRF2 levels across these cell lines relative to the internal control GAPDH. We found that levels of UHRF2 mRNA were comparably high in the RS4:11, SEMK2, and NALM6 cell lines but greatly reduced in Jurkat, K562 and RAJI cell lines. We saw a corresponding relationship between mRNA and protein levels from these cell lines, with Jurkat, K562 and RAJI cells not expressing UHRF2, and the RS4:11, SEMK2, and NALM6 lines highly expressing UHRF2 protein. UHRF2 protein was also reduced in U2OS cells stably integrated with an shRNA against UHRF2, which we have previously described [34]. These findings indicate that just as for human solid tumors, UHRF2 expression is severely reduced in some hematopoietic malignancies.

**Figure 6:**
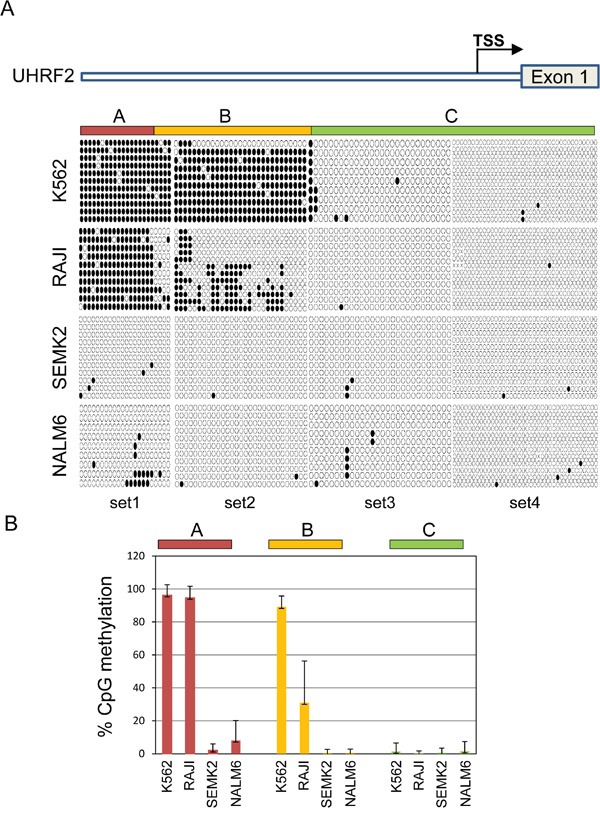
The UHRF2 promoter is significantly methylated in non-expressing cell lines **A.** Human UHRF2 genomic locus and CpGs analyzed by bisulfite sequencing. Bisulfite sequencing results of individual amplicons are represented by rows of circles. Solid circles represent methylated CpGs and open circles denote unmethylated CpGs. **B.** Methylation status of UHRF2 promoter in hematopoietic cancer cell lines. The percent of CpGs methylated in regions “A”, “B”, and “C” were quantified and graphed. Error bars represent standard deviation from the mean.

Hypermethylation of CGIs in the promoter regions of genes is a frequent cause of tumor suppressor inactivation in human cancer. The UHRF2 promoter contains a 1744 nucleotide region predicted to be a CpG island (65.7% GC, 0.95 CpG observed [Obs]/global expected [Exp]) compared to the classic definition of a CpG island (GC > 50%, Obs/Exp > 0.6). We thought that differential hypermethylation of the UHRF2 promoter might reasonably explain its reduced levels in human solid and hematological malignancies. We tested this directly by measuring CpG methylation of the UHRF2 promoter from bisulfite-treated genomic DNA isolated from two cell lines that express UHRF2 (SEMK2 & NALM6) and two that don't (K562 and RAJI). We observed significant CpGs methylation of in the promoters of K562 and RAJI, compared to almost none in the SEMK2 and NALM6 cell lines (Figure 6A). Closer inspection of K562 and RAJI CpG methylation revealed two distinct regions of the promoter with different levels of methylation between these lines. One region, arbitrarily named “A” was almost fully methylated in both K562 and RAJI cell lines. A second region termed “B”, which spanned three PCR amplification regions, was almost 100% methylated in K562 but the proportion of CpG methylation dropped to around 50% across this region in RAJI cells. A final region named “C” was unmethylated in each of the lines. Thus, loss of UHRF2 expression can be tied to promoter hypermethylation of CpGs in a leukemia and lymphoma cell line. The % CpG methylation was calculated for each region and cell line and displayed in Figure 6B.

**Figure 7:**
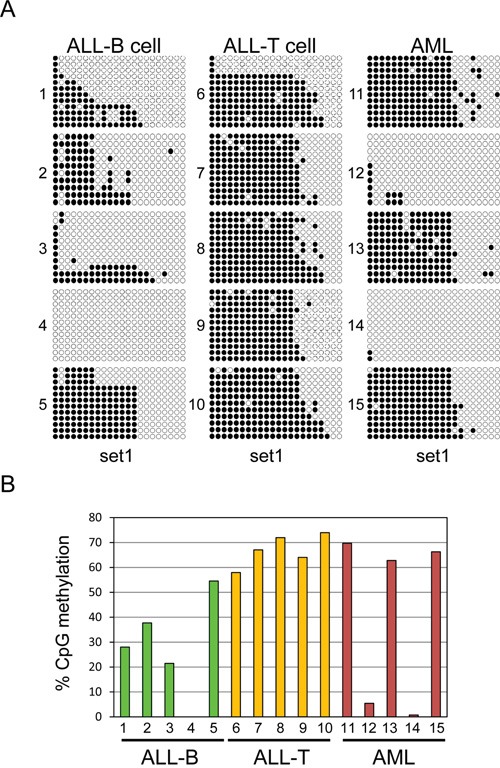
Variable UHRF2 methylation patterns in human leukemias **A.** Bisulfite sequencing of DNA from human ALL-B cell (5), ALL-T cell (5) and AML (5). Individual CpGs are represented by solid circles (methylated) or open (unmethylated). **B.** The percent of CpGs methylated across this region were quantified and graphed.

We examined methylation of UHRF2 in primary human leukemia cells to determine the cancer relevance of these findings. We obtained 5 B-cell ALLs, 5 T-cell ALLs, and 5 AMLs with high blast counts (>80%) and analyzed the genomic DNA by bisulfite sequencing. We used the primer set specific to the most 5′ region of the CpG island, which seemed to provide the best correlation to protein expression in the cell line analysis. Each of the different tumor types displayed a unique UHRF2 methylation pattern (Figure 7A). Three of the B-cell ALLs were methylated around 25% in this region, with another showing no methylation, and a final sample at 54% (Figure 7B). All of the T-cell ALLs were methylated across this region, averaging around 65%, and thus generally much more methylated than the B-cell ALLs. Of the AML samples, three closely resembled the 65% methylation pattern of the T-cell ALLs. The remaining two samples had less than 5% methylation. These finding indicate that the UHRF2 promoter is fairly heavily methylated in some cancer types but is greatly reduced in other types or individual cases.

**Figure 8:**
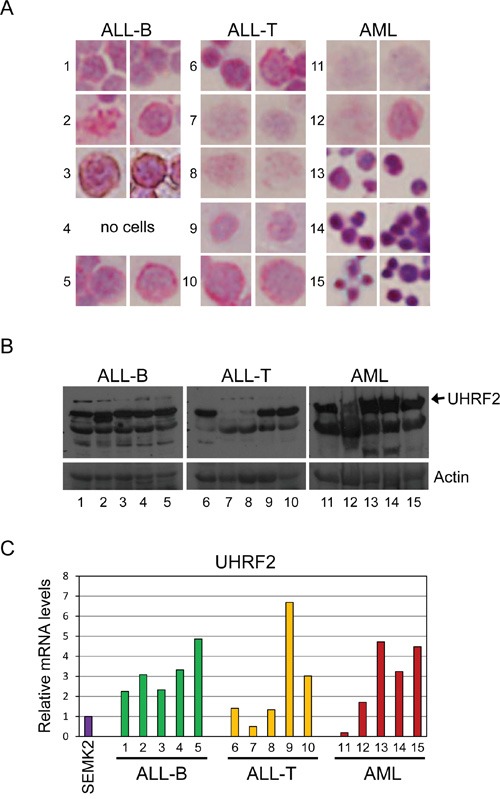
UHRF2 expression is altered in human leukemias The remaining cells were split for UHRF2 expression analysis. **A.** Tumor cells were centrifuged onto slides, fixed, and analyzed by IHC for UHRF2 (1:2000). **B.** Proteins from tumor cell lysates were analyzed by SDS-PAGE and immunoblotted for UHRF2 and ACTIN. **C.** UHRF2 mRNA levels in tumor cells assessed by qPCR.

We next determined if methylation patterns in the UHRF2 promoter correlated with protein expression levels in these samples. To do this, the remainder of the tumor samples were split into portions for anti-UHRF2 cell staining on slides (Figure 8A), for protein immunoblotting (8B), and mRNA analysis of UHRF2 levels by qPCR (8C). Anti-UHRF2 IHC staining of cells centrifuged onto slides was validated using cell lines that express high levels (SEMK2) or low levels (K562) of UHRF2 (Supplementary Figure S5). One of the ALL-B cell leukemias stainings lost cells on the slide and was not stained. The other 4 all stained positive for UHRF2 and apparent localization at heterochromatic foci as has been described [11, 12]. All 5 ALL-B cell leukemias expressed UHRF2 by immunoblotting, indicating a good agreement with the cytospin staining. Likewise, UHRF2 mRNA was high in each of these sample compared to levels in the SEMK2 cell line. These findings indicate that this region of the UHRF2 CpG island is poorly methylated in 4/5 ALL-B cell leukemias and this correlates with higher UHRF2 mRNA and protein levels. Among the ALL-T cell leukemias, which all were strongly methylated in this region, 2 of the 5 showed a reduction in UHRF2 protein levels by IHC and immunoblotting. However, the other samples still expressed UHRF2 protein and mRNA in spite of this region of the CpG island being hypermethylated. Similarly, AML samples were either fully methylated (3/5) or unmethylated (2/5). Of the 3 fully methylated, 2 still showed UHRF2 protein and mRNA expression. Of the 2 unmethylated, 1 showed strong UHRF2 protein and mRNA expression. These findings indicate that methylation of this region is alone insufficient to block UHRF2 expression.

## DISCUSSION

UHRF2 is the most closely related and structurally similar homolog of UHRF1, and both share some overlapping and unique characteristics [12, 13]. UHRF1 protein binds to hemi-methylated DNA during replication and recruits DNMT1 to copy the methyl pattern to the daughter strand [15–17]. Whereas the UHRF1 SRA domain shows strongest preference for hemi-methylated DNA, the SRA domain in UHRF2 show strongest biochemical affinity toward fully 5-hydroxymethylated cytosine [9, 10]. UHRF1 and UHRF2 also differ significantly in their expression patterns. UHRF1 is highly expressed in proliferating embryonic stem cells (HSCs) and a wide variety of proliferating cancer cells, but not in differentiated mature populations. In contrast, UHRF2 is not expressed in ESCs but its mRNA expression rises as ESC differentiate [12, 35].

Our results indicate that UHRF2 protein levels are widely lost in human cancer, with significant protein level reductions occurring in gastric, liver, pancreatic, lymphoma, cervical, endometrial, squamous cell carcinoma, and head and neck cancers. In gastric and liver cancers, UHRF2 appears to be preferentially localized to the cytoplasm in a subset of these tumors. A similar staining profile was observed by Lu and colleagues, who analyzed UHRF2 staining in a large cohort of primary colorectal tumors [33]. They observed low UHRF2 levels in 34% of tumors, and strong UHRF2 staining in 37% of tumors, with the majority of UHRF2 localized to the cytoplasm in the strong staining tumors. The functional significance or function of cytoplasmic localized UHRF2, or what signaling events cause it to be excluded from the nucleus is unknown. However, the highly similar UHRF1 is also present in the nucleus and cytoplasm and its phosphorylation by CCNA2/CDK2 during S-phase promotes its cytoplasmic localization, and mutating the phosphor-acceptor site leads to UHRF1 nuclear accumulation [36]. Thus, altered cell cycle signaling in certain tumors may induce the cytoplasmic localization of UHRF2.

UHRF2 is altered in human tumors through a variety of mechanisms. The first, although rare, is through direct mutation in its coding sequence [21, 22]. Second, UHRF2 is located at 9p24 and is subject to DNA copy number loss in human brain, breast, gastric, kidney, hematopoietic, and lung tumors [20]. It is also overexpressed at the mRNA levels in lung adenocarcinomas and squamous cell carcinomas compared to normal tissue, although not as highly as UHRF1 or DNMTs [37]. Third, UHRF2 promoter is hypermethylated in cancer. Varley and colleagues analyzed promoter methylation of each of the 100 “CAN” genes identified as mutated in breast and colon cancer and found that UHRF2 was one of only 5 genes that was methylated in both tumor types but not normal matched tissue [38]. Our results have extended these findings by demonstrating that expression of UHRF2 mRNA is lost in cell lines with hypermethylated UHRF2. We also observe UHRF2 methylation patterns that are distinct for human T- ALL, B-ALL, and AMLs. We were unable to definitively tie methylation status of UHRF2 to its expression in human leukemia samples. However, it is important to point out that standard bisulfite sequence does not discriminate mC from hmC. Thus it is possible that our tumor samples with high UHRF2 expression have hydryxomethylated promoters which might cause increased gene expression even though they were detected as hypermethylated. Together, these findings indicate that UHRF2 and 5hmC are widely present in differentiated human tissues, and UHRF2 protein is poorly expressed or mislocalized in diverse human cancers.

## MATERIALS AND METHODS

### TMA construction & immunohistochemistry

TMAs were constructed from normal and neoplastic tissues collected at the University of Minnesota Medical Center. IHC was performed as previously described [39]. Briefly, detection was through primary antibodies against UHRF2 (Sigma HPA026697/HPA026633) (1:200), 5hmC (Active Motif F3165) (1:5000), & Ki-67 (SP6) (Biocare Medical, Cat# CRM325), with Vector biotinylated secondary (1:250), tertiary was streptavidin-horseradish peroxidase (Covance #SIG-32000) and chromagen 3, 3′-diaminobenzidine substrate (Covance #SIG-31043). Micrographs were taken at 400x magnification, except where noted.

### Cell culture, RNA isolation, real-time PCR, and protein immunoblotting

Cells were cultured as previously described [40]. RNA was isolated and quantitative PCR performed as described [41]. Immunoblotting was performed as described [42]. Antiserum against UHRF2 for immunoblotting was purchased from Sigma (HPA026633).

### Mature lymphocyte isolation

For T, B, NK and monocyte isolation, peripheral blood was obtained from healthy donors and red blood cells and granulocytes were removed by Ficoll density centrifugation. Mononuclear cells were isolated and subjected to magnetic bead separation using T, B, NK or monocyte isolation kits (#130-050-101, 130-092-657, 130-050-301, 130-050-201) according to the manufactures specifications (Miltenyi Biotech, Auburn, CA). Samples were >85% pure by FACS analysis.

### Hematopoietic stem and progenitor cell isolation

Umbilical cord blood mononuclear cells were isolated using Ficoll density centrifugation. CD34+ cells were isolated using magnetic bead selection according to the manufactures specification (#130-046-702, Miltenyi Biotech, Auburn, CA). Isolated cells were further purified using FACS sorting (FACS Aria I) into the following populations: HSC (CD34+CD38-CD90+CD45RA-), MPP (CD34+CD38-CD90-CD45RA-), CMP (CD34+CD38+CD135+CD45RA-) and CLP (CD34+CD10+). Purified cells were resuspended in RLT buffer for RNA isolation.

### Leukemia tumor sample analysis

5 cases each of human B-cell ALL, T-cell ALL, and AML (blast count > 75%) were obtained from the University of Minnesota Leukemia MDS Tissue Bank (IRB# 1603R86027). For cytospin analysis, 20,000 cells were spun onto a slide (10 min/1000 rpm) using a Shandon Cytospin 3 centrifuge. Cells were fixed with 10% formalin for 20 minutes at room temperature, washed three times with PBS before analyzing by IHC using anti-UHRF2 antisera (1:2000).

### Bisulfite DNA conversion and sequencing method

Genomic DNA was isolated from cell lines using QIAamp DNA Mini kit (Qiagen) according to the manufacturer's manual. Bisulfite conversion was performed using the EpiTect Fast DNA Bisulfite kit (Qiagen) following manufacturer's protocol. DNA Single-step PCR amplification was conducted using Accuprime supermix II (Invitrogen). Primers were designed to specifically amplify converted DNA using the publicly available EpiDesigner BETA (http://www.epidesigner.com) and amplification products were visualized by agarose gel electrophoresis and appropriate bands were purified by using QIAquick Gel Extraction kit (Qiagen). Purified products were cloned into Topo TA vector for sequencing. Alignment and methylation analysis were performed using the online QUMA program (http://quma.cdb.riken.jp).

Primers for bisulfite: set1: left: GATTTTTTAGTT GTAGTAGGGAAGGA, right: ACAACTCCAAACCTA TCCTCAAAC; set2: left: GGTTTGAGGATAGGTTT GGAGTT, right:AATTCTTTAATCTCAAAAACACACCA; set3:left:TGGTGTGTTTTTGAGATTAAAGAATTA, right:AAAACTAAAACTCCCACATAAAAATC; set4: left:TGATTTTTATGTGGGAGTTTTAGTTTT, right:CCCTTTATCTCCCCCTAAACTCTA

## SUPPLEMENTARY FIGURES


