# Supplementary material for: Loss of UHRF2 expression is associated with human neoplasia, promoter hypermethylation, decreased 5-hydroxymethylcytosine, and high proliferative activity
Source: Oncotarget. 2016 Oct 12;7(46):76047–61. doi: 10.18632/oncotarget.12583 (PMC5340178; doi:10.18632/oncotarget.12583)
Supplement: Supplementary file 1 [file oncotarget-07-76047-s001.pdf]

Loss of UHRF2 expression is associated with human neoplasia, promoter hypermethylation, decreased 5-hydroxymethylcytosine, and high proliferative activity

SUPPLEMENTARY FIGURES

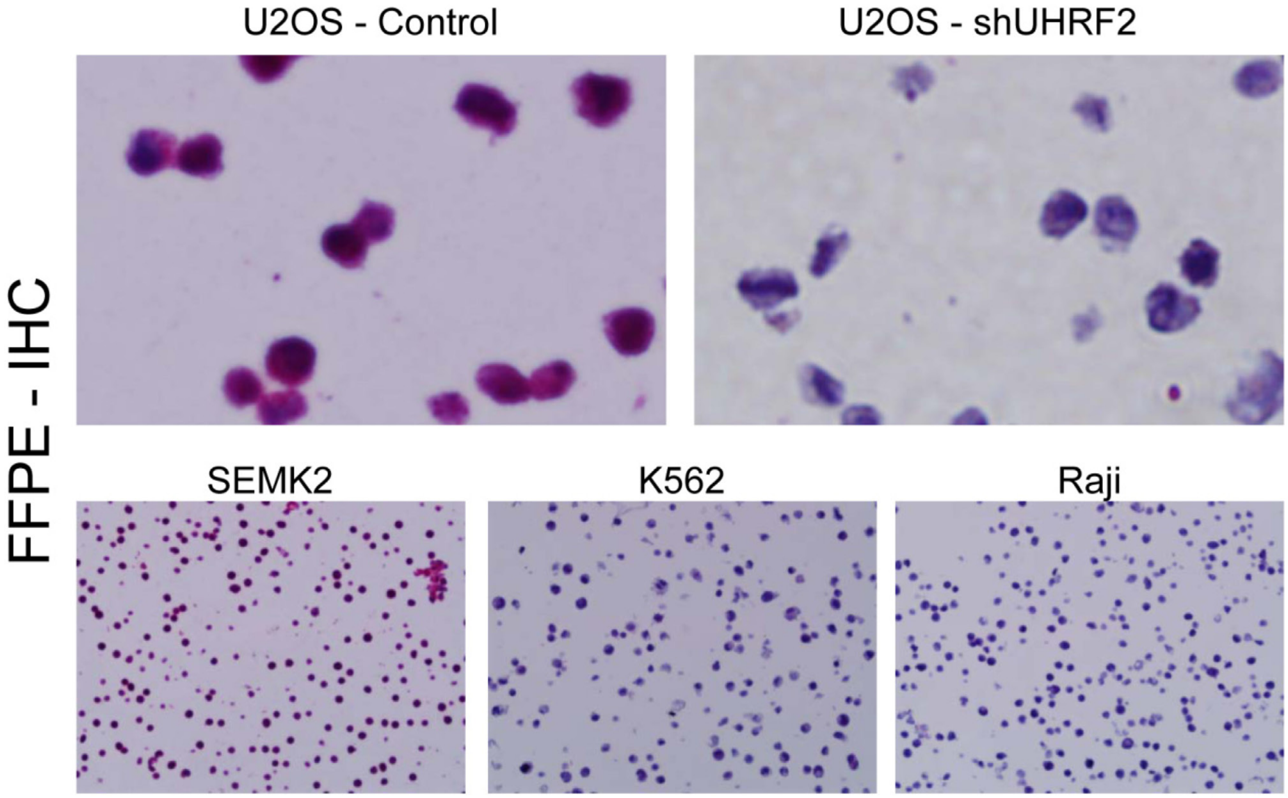

Supplementary Figure S1: IHC for UHRF2 was performed on cell lines (U2OS – control, U2OS – shUHRF2, SEMK2, K562, and Raji) that were formalin fixed, pelleted and embedded in paraffin slides for UHRF2 IHC @ 1:1000 primary.

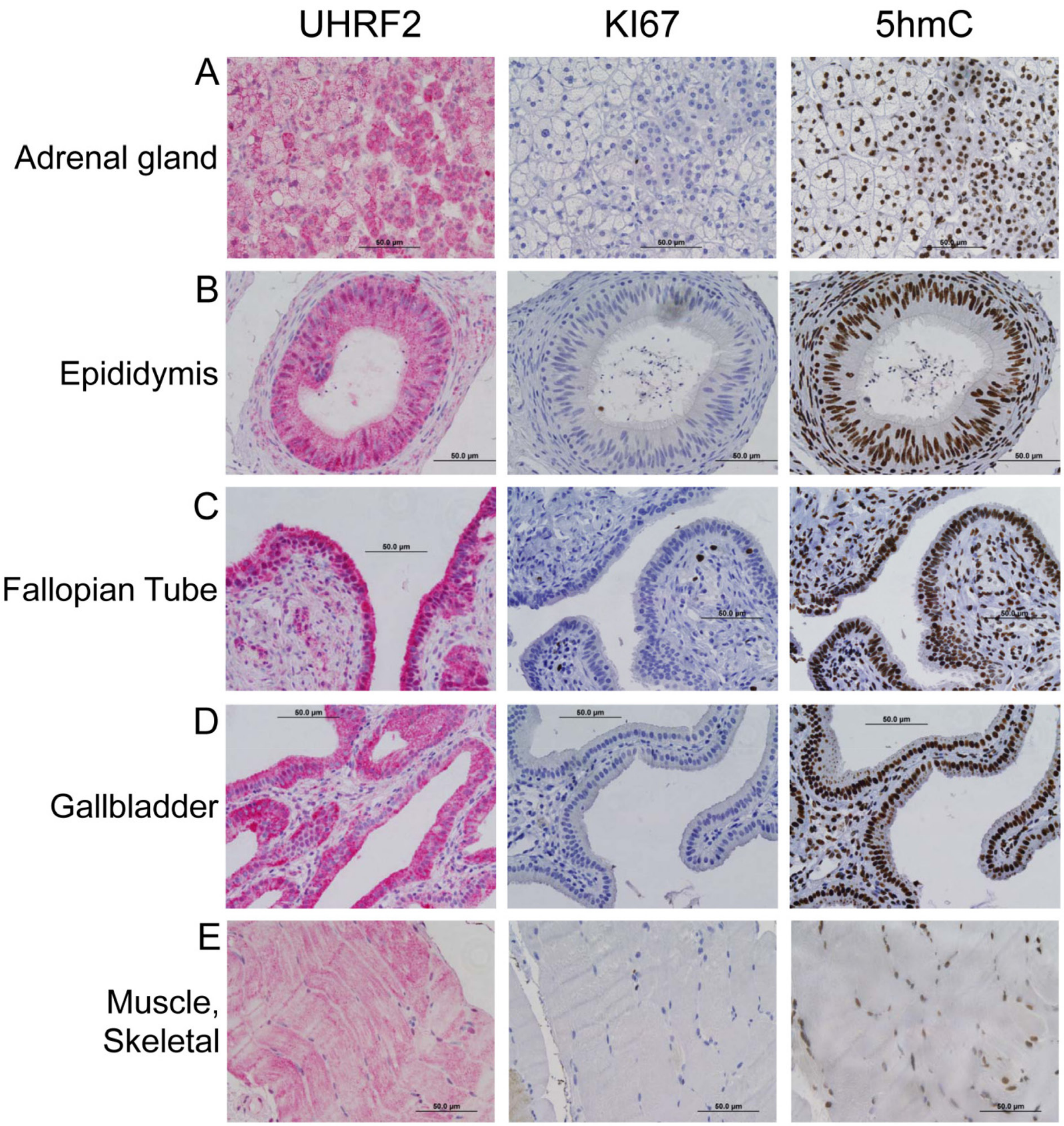

Supplementary Figure S2A: IHC for UHRF2, Ki67, and 5hmC was performed on normal human tissue from **A.** adrenal gland, **B.** epididymis,**C.** Fallopian tube, **D.** gallbladder, **E.** skeletal muscle.

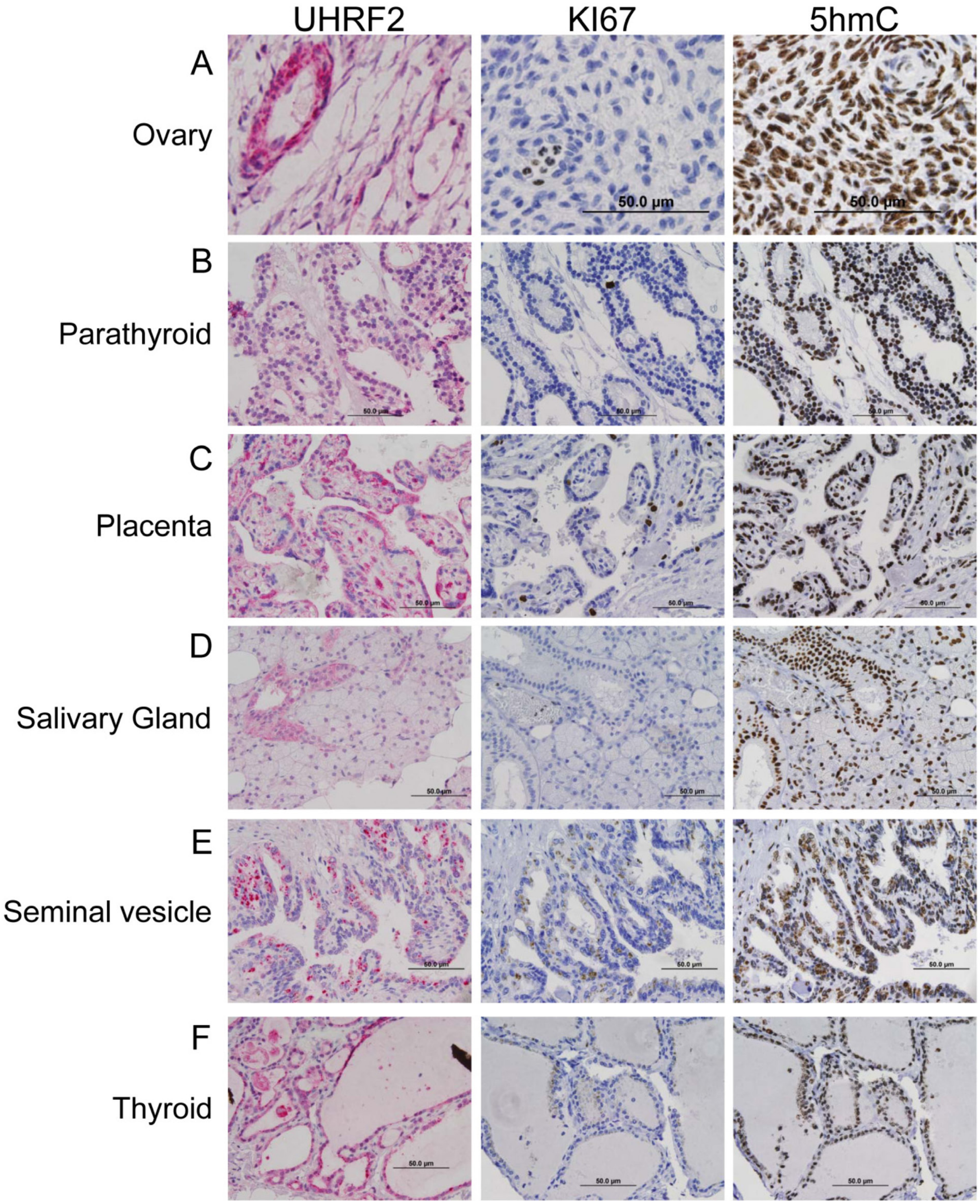

Supplementary Figure S2B: IHC for UHRF2, Ki67, and 5hmC was performed on normal human tissue from **A.** ovary, **B.** parathyroid, **C.** placenta, **D.** salivary gland, **E.** seminal vesicle, **F.** thyroid.

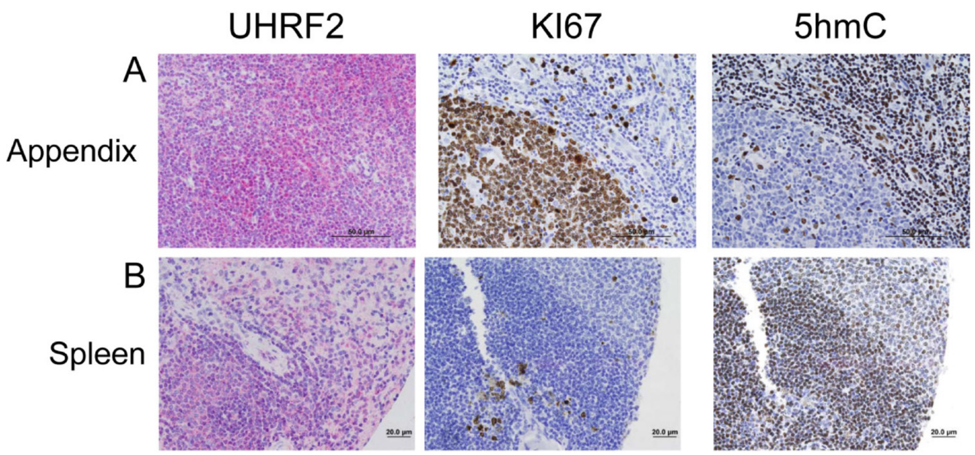

Supplementary Figure S3A: UHRF2, Ki67, & 5hmC staining in germinal centers from **A.** appendix and **B.** spleen.

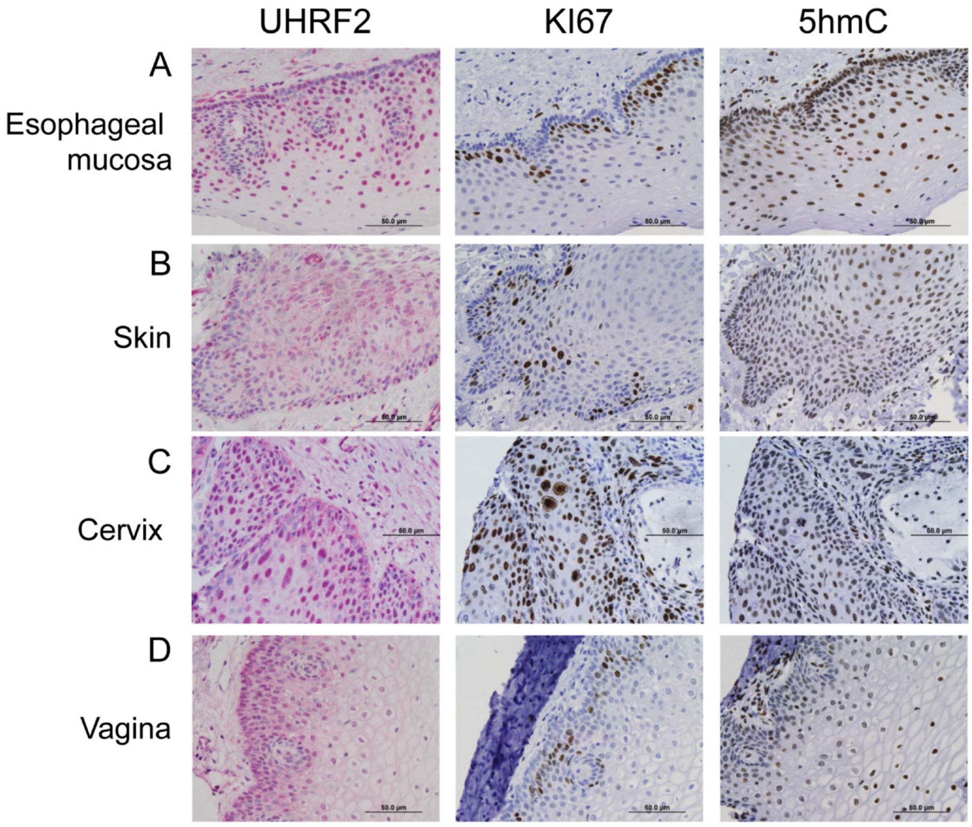

Supplementary Figure S3B: UHRF2, Ki67, & 5hmC staining in squamous epithelial cells from **A.** esophageal mucosa, **B.** skin, **C.** cervix, **D.** vagina.

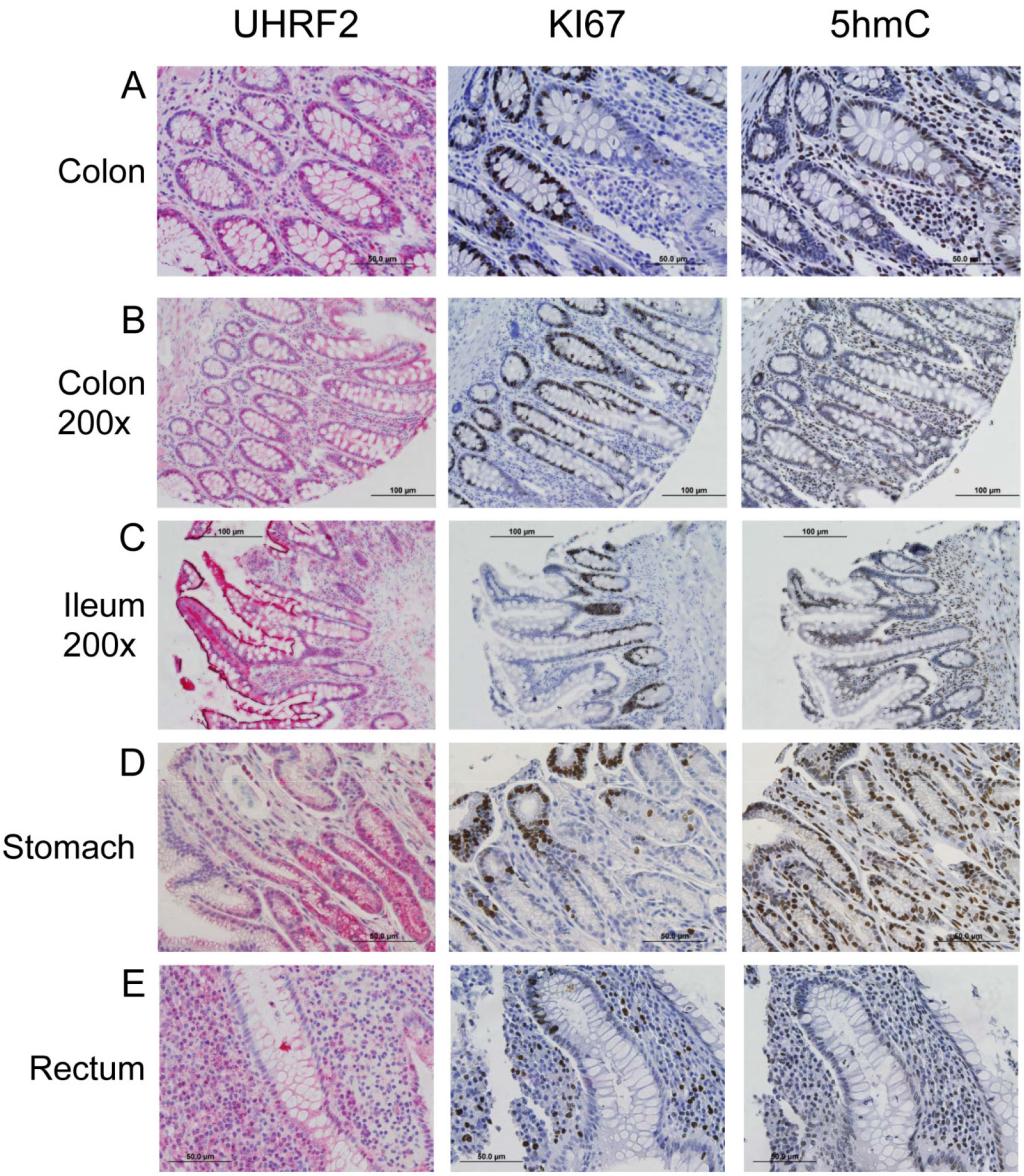

Supplementary Figure S3C: UHRF2, Ki67, & 5hmC staining in intestinal crypt and differentiating villi from **A.** colon, **B.** colon, **C.** ileum, **D.** stomach, **E.** rectum. B&D were taken at 200x magnification.

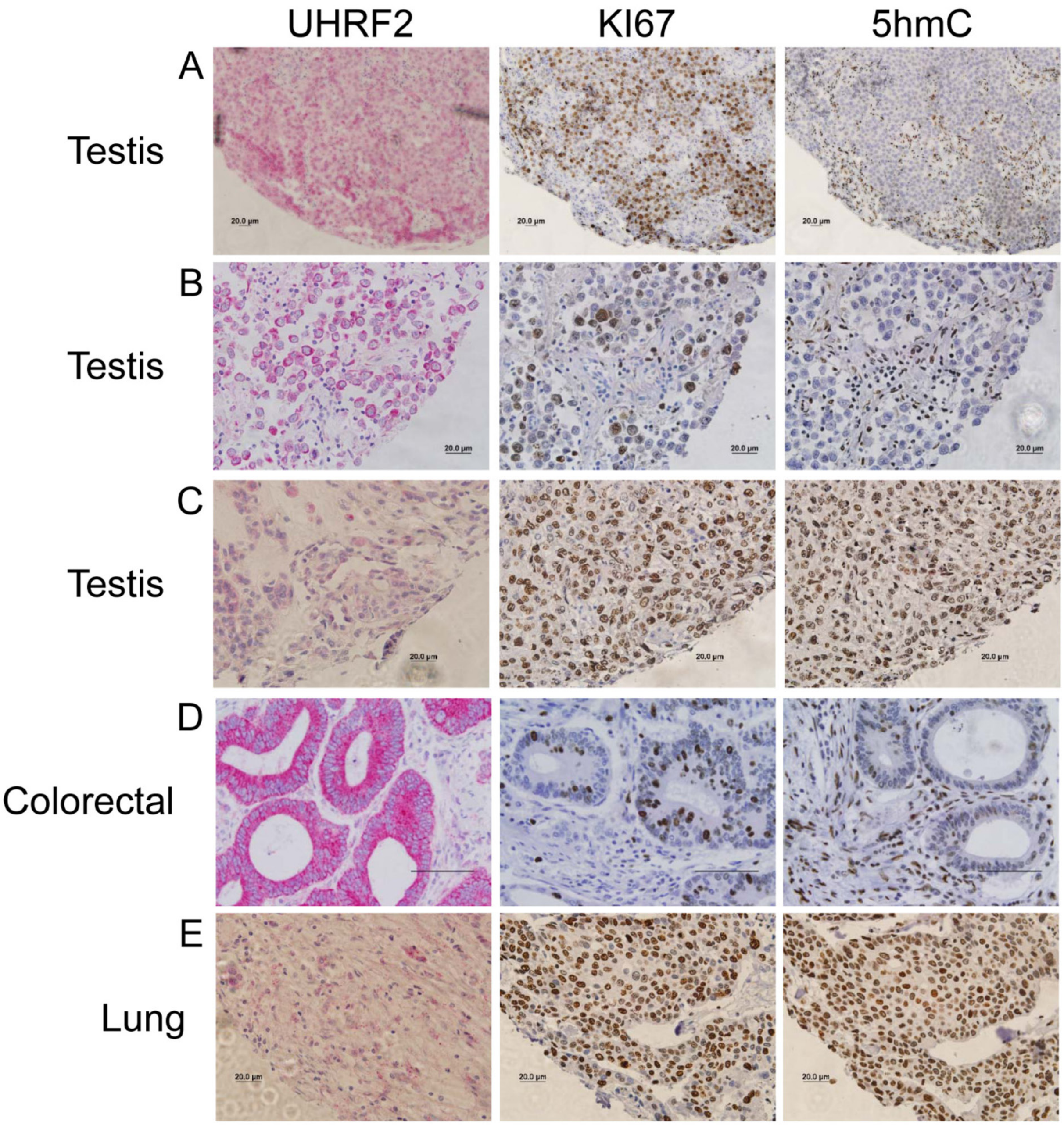

Supplementary Figure S4: A. UHRF2, Ki67, & 5hmC staining in cancer tissue originating from the **A-C.** testis, **D.** colon, or **E.** lung.

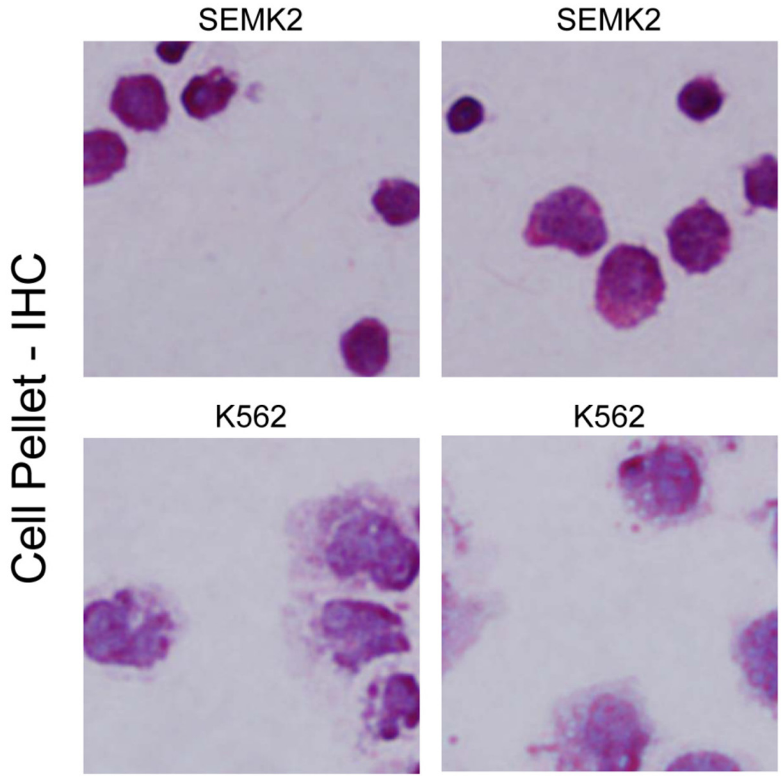

Supplementary Figure S5: IHC for UHRF2 was performed on K562 and SEMK2 cell lines that were centrifuged onto slides for UHRF2 IHC @ 1:2000 primary.
